# Fermentation Kinetics of Selected Dietary Fibers by Human Small Intestinal Microbiota Depend on the Type of Fiber and Subject

**DOI:** 10.1002/mnfr.202000455

**Published:** 2020-09-29

**Authors:** Mara P. H. van Trijp, Christiane Rösch, Ran An, Shohreh Keshtkar, Madelon J. Logtenberg, Gerben D. A. Hermes, Erwin G. Zoetendal, Henk A. Schols, Guido J. E. J. Hooiveld

**Affiliations:** ^1^ Nutrition, Metabolism and Genomics Group Division of Human Nutrition and Health Wageningen University Stippeneng 4 Wageningen WG 6708 The Netherlands; ^2^ Laboratory of Food Chemistry Wageningen University Bornse Weilanden 9 Wageningen WG 6708 The Netherlands; ^3^ Laboratory of Microbiology Wageningen University Stippeneng 4 Wageningen WG 6708 The Netherlands

**Keywords:** fiber, in vitro fermentation, microbiota, prebiotics, small intestine

## Abstract

**Scope:**

An underexplored topic is the investigation of health effects of dietary fibers via modulation of human small intestine (SI) microbiota. A few previous studies hint at fermentation of some dietary fibers in the distal SI of humans and pigs. Here the potential of human SI microbiota to degrade dietary fibers and produce metabolites in vitro is investigated.

**Methods and Results:**

Fructans, galacto‐oligosaccharides, lemon pectins, and isomalto/malto‐polysaccharides are subjected to in vitro batch fermentations inoculated with ileostomy effluent from five subjects. Fiber degradation products, formation of bacterial metabolites, and microbiota composition are determined over time. Galacto‐ and fructo‐oligosaccharides are rapidly utilized by the SI microbiota of all subjects. At 5h of fermentation, 31%–82% of galacto‐oligosaccharides and 29%–89% fructo‐oligosaccharides (degree of polymerization DP4‐8) are utilized. Breakdown of fructo‐oligosaccharides/inulin DP ≥ 10, lemon pectin, and iso‐malto/maltopolysaccharides only started after 7h incubation. Degradation of different fibers result in production of mainly acetate, and changed microbiota composition over time.

**Conclusion:**

Human SI microbiota have hydrolytic potential for prebiotic galacto‐ and fructo‐oligosaccharides. In contrast, the higher molecular weight fibers inulin, lemon pectin, and iso‐malto/maltopolysaccharides show slow fermentation rate. Fiber degradation kinetics and microbiota responses are subject dependent, therefore personalized nutritional fiber based strategies are required.

## Graphical abstract

An underexplored topic is the investigation of health effects of dietary fibers via modulation of bacteria residing inside the small intestine (SI). The authors study the interaction between multiple types of fibers and the human small intestine microbiota in vitro. Fermentation kinetics are dependent on the type and size of fiber, more “complex” dietary fibers are not degraded by SI microbiota.

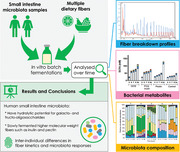

## 1 Introduction

Currently there is a strong interest in optimizing human health through the consumption of dietary fibers, due to their direct and indirect health benefits.^[^ ^1^, ^2^, ^3^ ^]^ Dietary fibers are present as natural constituents of leguminous seeds, fruits, vegetables, and cereals. Per definition, they resist hydrolysis by host digestive enzymes in the small intestine (SI),^[^ ^4^ ^]^ and some fibers can be fermented by the human intestine microbiota.^[^ ^5^ ^]^ During fermentation there is formation of for example glycosidic degradation products, and fermentation end products like short‐chain fatty acids (SCFA).^[^ ^6^ ^]^ SCFAs have been suggested to play a key role in the prevention and treatment of metabolic syndrome.^[^ ^7^, ^8^ ^]^ Soluble galacto‐oligosaccharides (GOS), fructans including inulin and fructo‐oligosaccharides (FOS), and more complex fibers such as pectins, and a novel fiber type isomalto/malto‐polysaccharides (IMMP), are known to stimulate the growth of a number of microbial species in the intestinal tract such as *Bifidobacterium* spp.^[^ ^9^, ^10^ ^]^ Part of the health benefits of fibers are thought to be mediated by changing the gut microbiota composition and/or activity. Although the bacterial load is highest in the large intestine, also considerable numbers of bacteria are present in the human distal SI, namely 10^7–8^ bacteria per gram of intestinal content versus 10^11^ bacteria number per gram in colonic content.^[^ ^11^ ^]^

An emerging field of research focusses on the interaction between the diet and the SI microbiota. The SI microbiota is likely very responsive to dietary perturbations, such as dietary lipids.^[^ ^12^, ^13^, ^14^ ^]^ Some dietary fibers, such as pectins, have the potential to directly activate the immune system in the SI, as was shown before in vitro,^[^ ^15^ ^]^ but an underexplored field is their potential indirect effects via modulations of SI microbiota. The microbiota can affect host metabolism and health through for instance excretion of signaling molecules that effect glucose homeostasis modulators.^[^ ^12^, ^16^ ^]^ Previous studies hinted at fermentation of some dietary fibers in the distal SI of humans and pigs,^[^ ^17^, ^18^, ^19^, ^20^ ^]^ showing the potential health impact of fiber via microbiota residing in the upper intestinal tract. Ileostomy effluent microbiota was previously found to resemble the microbiota as found in the jejunum and ileum of healthy subjects, and can therefore be used as a model to study the human SI microbiota.^[^ ^21^ ^]^

Since there is limited knowledge on the capability of human SI microbiota to metabolize dietary fibers, we investigated in an explorative way the potential of individual human SI microbiota to break down dietary fibers and produce metabolites of interest to health in vitro. We performed in vitro batch fermentations with human ileostomy microbiota with FOS/inulin, GOS, and the complex high molecular weight fibers lemon pectin, and IMMP.

## 2 Results

### 2.1 Subject Characteristics, Dietary Intake, and Ileostomy Effluent

The five ileostomy subjects had an age range of 30–75 years, a mean BMI of 21.1 ± 4.8 kg m^−2^, included two males and three females, and the years of ileostomy wearing ranged between 1–14 years (Table S1, Supporting Information). They were not using medication, or medication unrelated to intestinal disease, except for anti‐constipation drugs used by I1 (Table S1, Supporting Information). The two consecutive days before ileostomy effluent donation, I1, I2, and I5 consumed on average 28 ± 0.6, 20 ± 3.4, and 21 ± 2.7 gram dietary fibers per day, whereas I3 and I4 consumed 16 ± 1.6, and 13 ± 1.8 gram dietary fibers per day, respectively (Table S2, Supporting Information). The microbiota dataset was of sufficient quality (Figure S1, Supporting Information). The microbiota profiles in the ileostomy effluents used in this study showed high variation (*R* = 0.37 ± 0.22) among subjects, and included taxa that are often found in the human SI (Figure S2, Supporting Information).

### 2.2 Breakdown Kinetics of GOS

**Figure 1:**
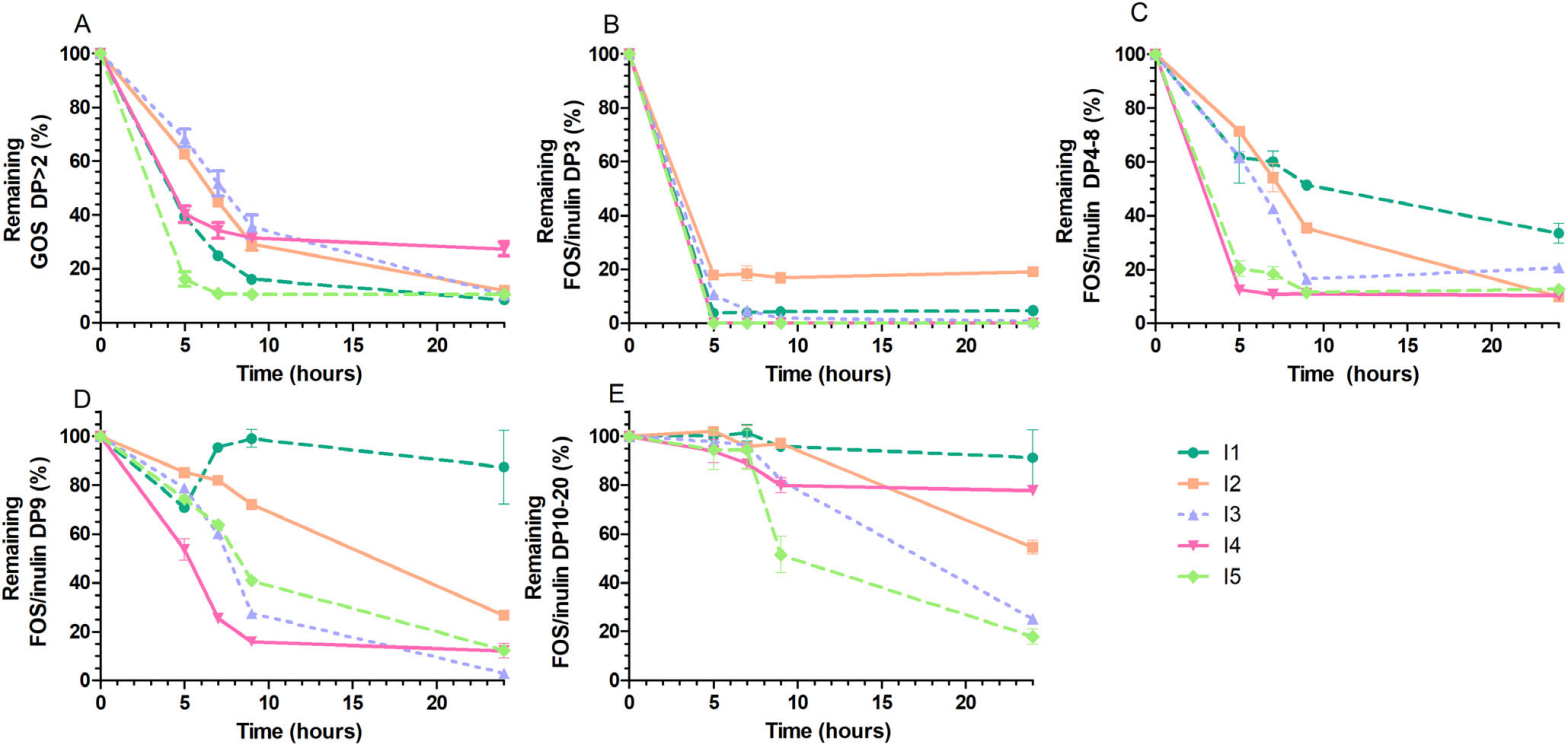
Degradation kinetics of GOS and FOS/inulin by SI microbiota. A) Degradation of GOS DP>2, B) FOS/inulin DP3, C) FOS/inulin DP4‐8, D) FOS/inulin DP9, and E) FOS/inulin DP10‐20 during fermentation at 0, 5, 7, 9, and 24 h. The lines represent the five subjects (I1‐I5). Degradation is expressed as percentage remaining from the initially present oligomers in the substrate. Values are means ± SDs, *n* = 2 technical replicates per subject.

Degradation of GOS, a well‐known soluble prebiotic, by SI microbiota was investigated (**Figure** **1**A). GOS with a degree of polymerization (DP) >2 were quickly utilized by microbiota of all SI samples (Figure 1A). After 5 h of incubation, breakdown of GOS varied from 31% (I3) to 84% (I5), and the amount of GOS decreased further over time from 5 to 9 h. In all incubations, small amounts of GOS were remaining after 24 h (8%–24%). In the control fermentations without added fibers, no GOS was detected. Overall, GOS was degraded by the SI microbiota of all individuals, with differences in kinetics mainly before 7 h, and always a small remainder GOS was left at 24 h.

### 2.3 Breakdown Kinetics of FOS/inulin

Degradation of FOS/inulin, also well‐known soluble prebiotics, by SI microbiota was studied (Figure 1B–D). The DP cut‐off values of degradation of oligomers in the FOS/inulin mixture were based on the breakdown kinetics by the SI samples, from fast (DP3, Figure 1B) to slower (DP4‐8, Figure 1C, and DP9, Figure 1D), or slowest or no breakdown at all (DP10‐20, Figure 1E). Oligomers present in FOS/inulin (DP3, DP4‐8) were utilized quickly by SI samples of all subjects (Figure 1B,C). The utilization of DP3 at 5 h varied from 100% (I4, I5) to 82% (I2), and the utilization of DP4‐8 at 5 h varied from 29% (I2) to 88% (I4). For all samples the breakdown of FOS/inulin DP10‐20 breakdown was negligible before 7 h (Figure 1D). I1 and I4 displayed no capacity to break down FOS/inulin DP10‐20, whereas the breakdown of DP10‐20 was observed in I2, I3, and I5, typically after 7–9 h when the DP4‐8 fraction was mostly utilized. In the control fermentations without added fibers, no FOS/inulin was detected. Taken together, fermentation kinetics of FOS/inulin was dependent on the chain size of the present molecules, and FOS/inulin DP3 and DP4‐8 degradation by SI microbiota were fast.

### 2.4 Breakdown Kinetics of Lemon Pectin

**Figure 2:**
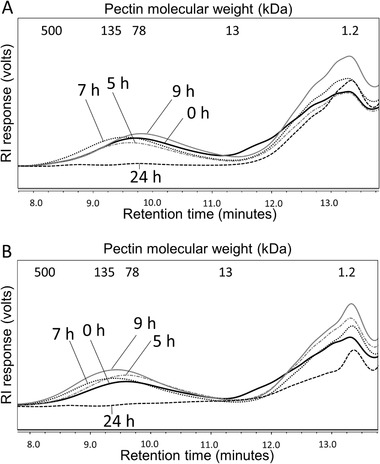
Degradation kinetics of lemon pectin by SI microbiota. Degradation of lemon pectin by two subjects A) I1 and B) I2 during fermentation at 0, 5, 7, 9, and 24 h. The indicated molecular weight in kDa was based on pullulan standards. The lemon pectin has a molecular weight distribution of 15–500 kDa. The lower MW components of 1.2–13 kDa originate from the ileostomy effluent and SIEM medium. Technical replicates are not shown.

Besides GOS and FOS/inulin, a high molecular weight fiber was studied, namely lemon pectin. Changes in the water‐soluble lemon pectin abundance during fermentation as monitored by HPSEC was only studied using I1 and I2 (**Figure** **2**). The molecular weight (MW) distributions of the lemon pectin (13–500 kDa) revealed that pectin was slowly degraded by the SI microbiota, since no degradation of pectin by I1 (Figure 2A) and I2 (Figure 2B) was observed until 9 h. After 24 h, the lemon pectin 13–500 kDa was completely utilized in both I1 and I2. Since lemon pectin was very slowly degraded by I1 and I2, another fiber was therefore selected for use in subsequent experiments with I3, I4, and I5 to investigate whether this was also the case for another type of high complexity fiber with a different backbone and linkages, namely IMMP.

### 2.5 Breakdown Kinetics of IMMP

**Figure 3:**
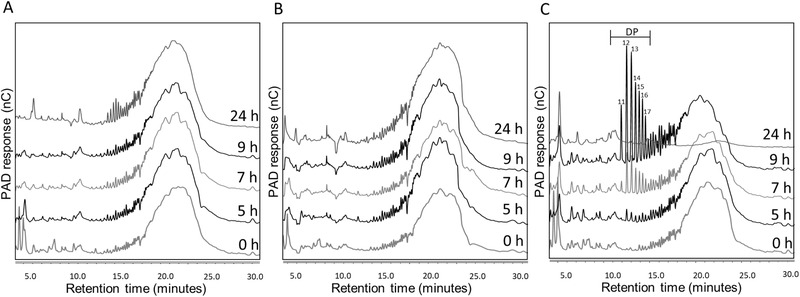
Degradation kinetics of IMMP by SI microbiota. Degradation of IMMP in vitro by three subjects A) I3, B) I4, and C) I5 during fermentation at 0, 5, 7, 9, and 24 h. Different DPs of IMMP are annotated. Larger saccharides elute later from the column, and appear more to the right side of the HPAEC‐PAD chromatogram. Technical replicates are not shown.

IMMP fermentation (**Figure** **3**) was studied using I3 (Figure 3A), I4 (Figure 3B), and I5 (Figure 3C). During IMMP fermentation, only negligible amounts of isomalto‐oligosaccharides (IMO), previously found to have α‐1,6‐glycosidic linkages,^[^ ^10^ ^]^ appeared at 24 h by I3 (Figure 3A). No IMMP breakdown was observed by I4 over 24 h time (Figure 3B). In contrast, IMMP was degraded by I5 between 7 and 24 h, since IMO with DP11‐23 became apparent at 7 and 9 h (Figure 3C), and at 24 h the unseparated polysaccharide fraction and the IMO were completely utilized. Overall, IMMP was not degraded by I3 and I4, and slowly degraded by I5.

### 2.6 Formation of Microbial Fermentation Products

**Figure 4:**
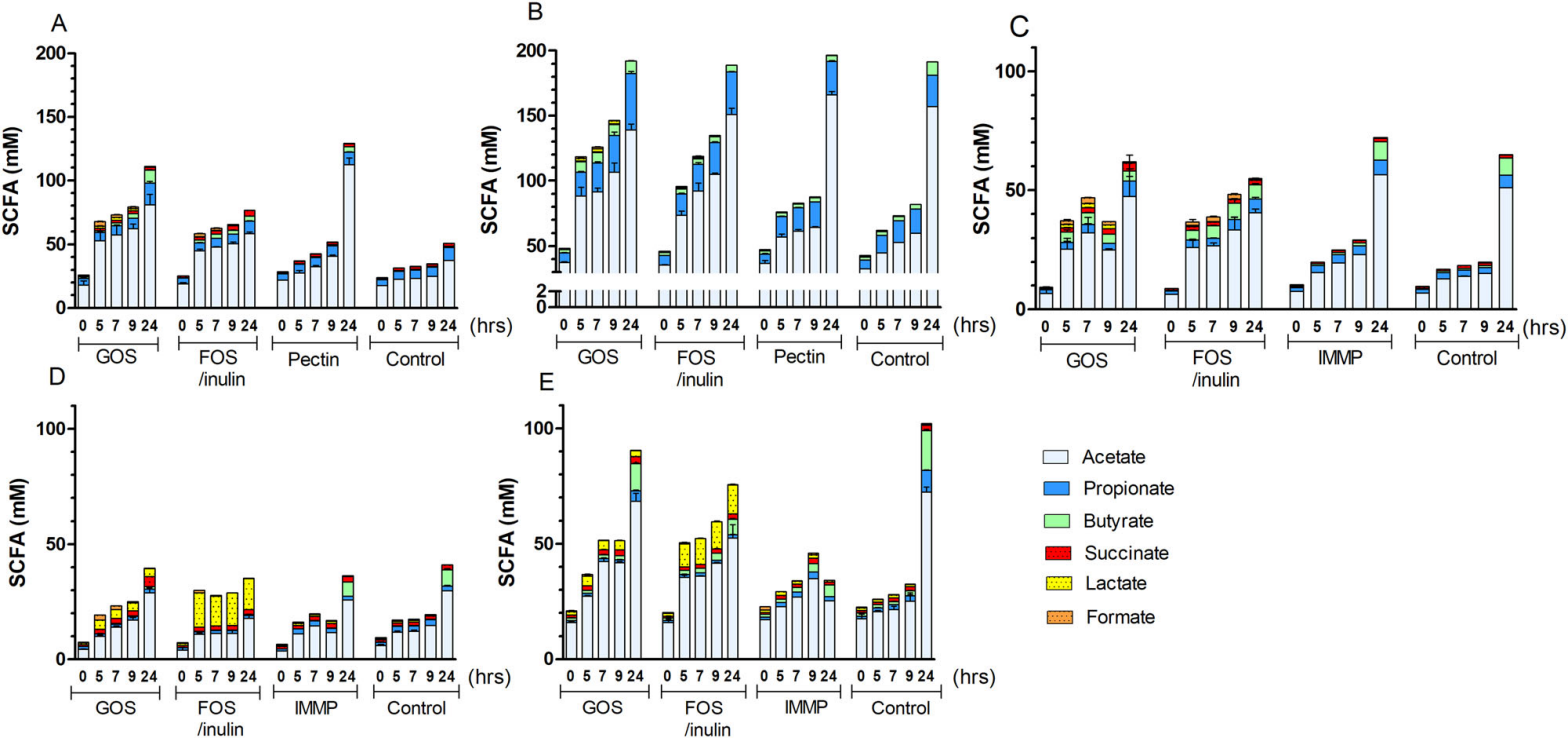
Production of bacterial metabolites by SI microbiota. SCFA concentrations during fermentation of different fibers at 0, 5, 7, 9, and 24 h by five subjects A) I1, B) I2, C) I3, D) I4, and E) I5. Values are means ± SDs, *n* = 2 technical replicates per subject.

The main microbial fermentation metabolites acetate, propionate, butyrate and lactate, formate, succinate were measured as an indicator of fiber fermentation (**Figure** **4**). Independent of the type of fiber, activity of SI microbiota was mostly reflected by increased acetate concentrations over time (*p*‐values < 0.05). FOS/inulin and GOS increased propionate concentrations at 5, 7, and 9 h compared to control (*p*‐values < 0.05) when taken the five subjects together (Figure 4A–E). FOS/inulin also increased lactate formation at 5, 7, and 9 h (*p*‐values < 0.05) compared to control. GOS increased lactate formation at 7 and 9 h (*p*‐values < 0.05) compared to the control. Lactate concentrations were increased mostly in FOS/inulin and GOS fermentation samples of I4 (Figure 4D) and I5 (Figure 4E). The increased metabolite concentrations during GOS and FOS/inulin fermentation before 9 h (Figure 4) were reflected by decreased pH values (Figure S3, Supporting Information) compared to controls.

Lemon pectin (Figure 4A,B) and IMMP (Figure 4C–E) generated limited fermentation products also mentioned at the end of the sentence before 9 h (*p*‐values > 0.05) compared to control. This was in line with a slow fiber breakdown, which started only after 9 h, or not at all (Figures 2, 3). The total metabolite concentrations in the controls without added fiber increased mainly between 9 and 24 h, and therefore at 24 h no statistical comparisons between control and fiber were made. Overall, FOS/inulin and GOS, whereas not IMMP and pectin, significantly increased the concentrations of some SCFAs before 9 h compared to control without added fibers.

### 2.7 Microbiota Diversity during Fermentation of Fibers

**Figure 5:**
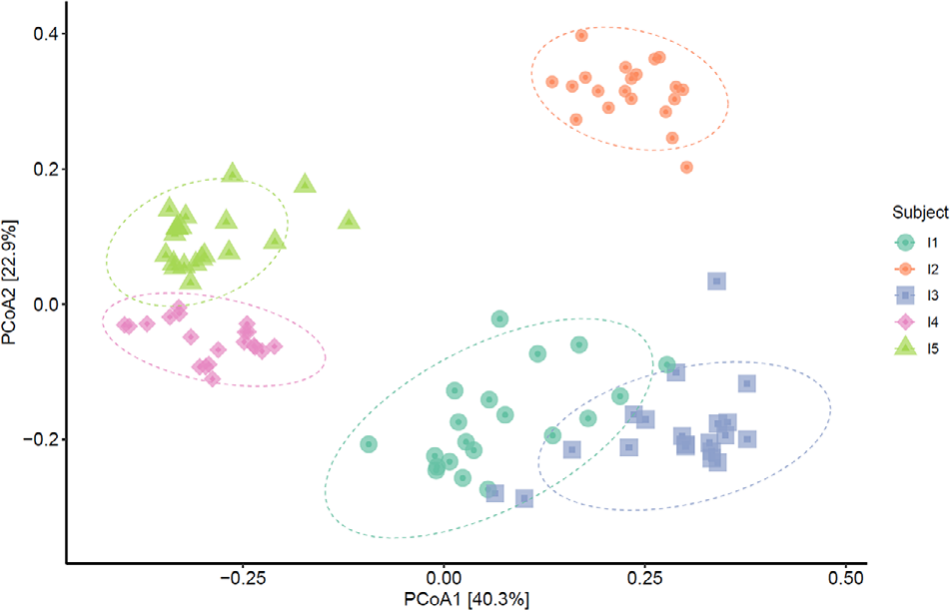
Overall microbiota differences (beta‐diversity) in the in vitro fermentation dataset. PCoA plots to visualize microbiota variation between five different subjects (I1‐I5), where 95% confidence ellipses are shown.

Subsequently, the impact of fibers on the microbiota composition was studied. Visualization of beta‐diversity in microbiota composition during fiber fermentations revealed main clusters based on individuals (**Figure** **5**, PERMANOVA *p*‐value = 0.001), the subjects explained 70% of the variation in the dataset (*R* ^2^ subject = 0.70). The bacterial communities in the fermentation samples from different subjects were all different from each other (PERMANOVA *p*‐values < 0.05). The results in the PCoA plot are represented in a heatmap, visualizing the specific bacteria causing differences between the fermentation samples (Figure S4, Supporting Information). A smaller fraction of variation was explained by fiber type (9%, PERMANOVA *p*‐value = 0.002, *R* ^2^ fibers = 0.090) and the fermentation time point (2%, PERMANOVA *p*‐value = 0.06, *R* ^2^ time = 0.023). Furthermore, the microbiota alpha‐diversity over time was increased by FOS/inulin in I2 and I3 and by GOS in I2 when compared to controls (Figure S5, Supporting Information). Pectin did not increase alpha‐diversity when compared to control, and IMMP increased alpha‐diversity after 7 h in I5 when compared to control. The microbiota dissimilarity between fermentation reactions with fibers compared to controls without fiber (Figure S6, Supporting Information) revealed that GOS and FOS/inulin over time caused a more dissimilar microbiota composition in I1 (Figure S6A), I3 (Figure S6C), and I5 (Figure S6E). FOS/inulin, but not GOS, caused a dissimilar microbiota in I2 (Figure S6B) when compared to their controls. Pectin did not change the microbiota in I1 compared to control (Figure S6A), but in I2 it changed the microbiota composition after 9 h compared to control (Figure S6B). IMMP did not change the microbiota profile in I3 and I4, but it was changed after 7 h in I5, compared to their own controls (Figure S6C–E).

### 2.8 Microbiota Composition during Fiber Fermentation

**Figure 6:**
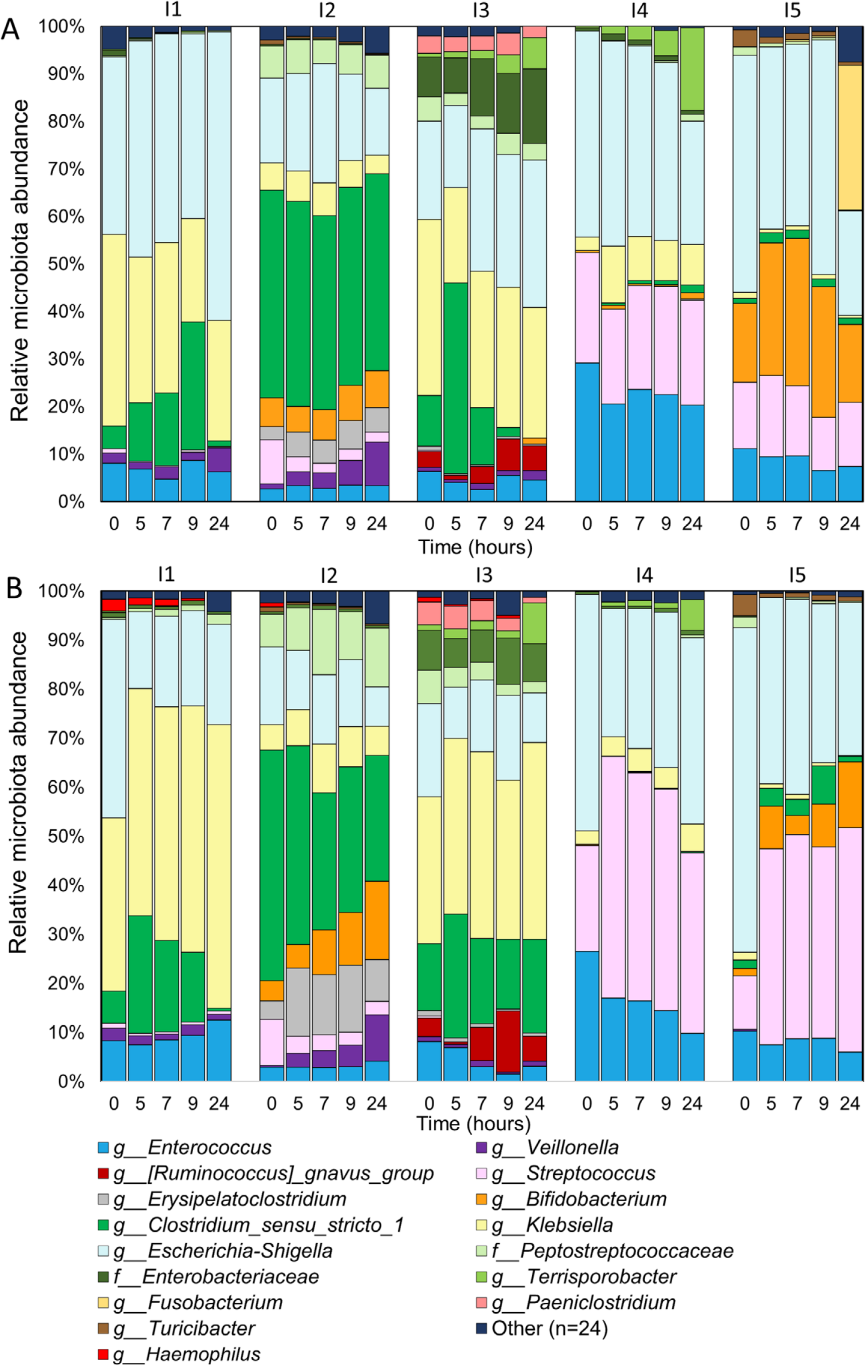
SI microbiota composition during GOS and FOS/inulin fermentation. Microbiota relative abundance of the top 15 genera (or highest known taxonomy) at 0, 5, 7, 9, and 24 h during in vitro fermentation of A) GOS and B) FOS/inulin by microbiota from the five subjects (I1‐I5).

To investigate microbiota changes over time, the relative microbiota composition after addition of GOS and FOS/inulin (**Figure** **6**), and lemon pectin and IMMP (Figure S7, Supporting Information) was plotted over time. Preselection of ileostomy effluent in medium caused a relative increase of *Escherichia‐Shigella* and *Klebsiella* (Enterobacteriaceae) at the start of the fermentation (Figure 6), compared to the microbiota composition in the ileostomy effluent (Figure S2, Supporting Information). GOS increased abundance of *Clostridium cluster 1* in I1 and I3 between 0 and 9 h (Figure 6A) when compared to the changes in microbiota in their controls (Figure S8, Supporting Information). GOS was degraded by I2 and I4, but minor microbiota composition changes were found over time. GOS increased *Bifidobacterium* abundance (from 16% to 28%) between 0 and 5 h in I5, and when GOS was utilized, *Fusobacterium* abundance (from 0.34% to 31%) increased between 9 and 24 h. FOS/inulin increased abundance of *Clostridium cluster_I* and decreased *Escherichia‐Shigella* upon fermentation by I1 (Figure 6B), and increased *Bifidobacterium*, *Veillonella*, and *Erysipelatoclostridium* over time in I2, compared to their controls. FOS/inulin did not selectively influence the microbiota in I3, whereas FOS/inulin increased. *Streptococcus* between 0 and 5 h in both I4 and I5, together with increased *Bifidobacterium* in I5. Lemon pectin did not modify the microbiota in I1 (Figure S7A, Supporting Information), but increased abundance of *Cellulosilyticum* in I2 between 9 and 24 h compared to their controls. IMMP increased *Bacteroides* and *Bifidobacterium* after 7 h (Figure S7B, Supporting Information) in I5 compared to control.

### 2.9 Changes in Total Bacterial Number

Indications of the total bacteria were generated by quantification of total bacterial 16S rRNA gene copy numbers in the fermentation samples over time (Figure S9, Supporting Information). Compared to control, GOS or FOS/inulin increased the total 16S rRNA gene copy numbers on average 1.74 ± 0.68 times or 2.50 ± 1.04 times at 9 h, respectively, whereas pectin or IMMP increased the copy numbers on average 1.38 ± 0.52 or 1.46 ± 0.63 times compared to control at 9 h, respectively.

## 3 Discussion

We investigated the potential of human SI microbiota to degrade dietary fibers in vitro, using ileostomy effluent samples. We showed a capacity of SI microbiota from five subjects to degrade GOS and FOS/inulin (DP3‐9), which started already before 5 h. Higher concentrations of metabolites were produced upon GOS and FOS/inulin fermentation compared to controls, which confirmed fiber fermentability. Although inclusion of only five subjects can be considered a weakness, in many in vitro fermentation studies fecal samples of different subjects are pooled ignoring individual variation. By using five individual SI samples in this study, we were able to show that despite different subject characteristics the SI microbiota from all individuals degraded GOS and FOS. In two studies that used metagenomics data it was hypothesized that SI microbes depend on capacity of rapid import and fermentation of available carbohydrates,^[^ ^21^ ^]^ and that human ileal mucosa bacteria are capable of breaking down GOS and FOS via expression of exo‐ and endo‐acting glycoside‐hydrolases,^[^ ^17^ ^]^ although ileum mucosa bacteria are not directly comparable to luminal microbiota. We found that FOS/inulin with a lower DP were hydrolyzed before longer‐chain FOS/inulin (DP10‐20), a preference also found for the colonic microbiota.^[^ ^22^ ^]^ Chain length preference is likely caused by expression of different microbial enzymes needed for optimal degradation of FOS (exo‐inulinases) and inulin (both exo‐ and endo‐inulinases), and carbohydrate transporters^[^ ^23^ ^]^ for their transportation.^[^ ^24^ ^]^ In a human ileostomy in vivo study it has been shown that lower molecular weight oligomers present in Jerusalem artichoke inulin (DP2‐60) were partly fermented (13%) in the SI, this breakdown was related to DP but unrelated to the transit time.^[^ ^25^ ^]^ A batch fermentation approach has some limitations, and therefore in vitro fermentation kinetics is not directly translatable to in vivo. The relative microbiota composition at the start of the fermentations differed from the microbiota composition in the ileostomy effluents. This compositional shift was likely caused by the preselection step in culture medium, which was applied to remove the remaining carbohydrates from the ileostomy effluent. The proteins and amino acids in this medium might have shifted the composition towards an increased abundance of protein‐fermenters such as Enterobacteriaceae, resulting in differences in microbial functionality.^[^ ^26^ ^]^ We have confirmed previous findings^[^ ^21^ ^]^ by detection of facultative and strict anaerobes in ileostomy effluent, but others also detected aerobic bacteria in the SI.^[^ ^27^ ^]^ The preparation procedure can impact bacteria cultivability and microbiota composition.^[^ ^28^ ^]^ By choosing anaerobic culturing methods, the SI microbiota composition possibly shifted towards a more anaerobic microbiota in vitro. This could influence the fermentation kinetics and increase SCFA production. However, bacteria known to be prebiotic utilizers, such as *Bifidobacterium*, *Lactobacillus* and *Streptococcus* ^[^ ^9^ ^]^ were already present in the initial microbiota composition of the ileostomy effluents, suggesting their growth can also by stimulated by prebiotics inside the SI. Moreover, concentrations of acetate, lactate, butyrate, and occasionally formate were found previously in ileostomy effluent, confirming the presence of SI bacteria with fermentative capacity.^[^ ^21^ ^]^ In sudden death victims only 13 ± 6 mm total SCFAs were measured in the terminal ileum, in contrast to 131 ± 9 in the proximal colon.^[^ ^29^ ^]^ The higher SCFA concentrations in our study might be explained by the (pre‐)incubation that increased the bacteria numbers, and bacteria numbers were estimated to be greater in ileostomy effluent than in the ileum of healthy subjects.^[^ ^30^ ^]^ Mainly acetate was produced by SI the microbiota upon fiber degradation in this study. In line with this finding, acetate production was higher during ileal fermentation compared to hindgut fermentation, whereas less butyrate was synthesized, in pigs fed a human‐type diet.^[^ ^18^ ^]^ Furthermore, considerable concentrations of acetate, but low concentrations of butyrate, were detected in ileal contents of pigs.^[^ ^31^ ^]^ The lower butyrate production by SI microbiota compared to colonic microbiota can be explained by a colon metagenome that was more enriched with the butyrate fermentation pathway compared to the SI microbiota metagenome.^[^ ^21^ ^]^ The presence of metabolites in the SI could have health implications, either locally in the intestine such as anti‐inflammatory effects,^[^ ^32^ ^]^ although mainly described for butyrate, or metabolic effects via uptake in the systemic circulation^[^ ^7^ ^]^ since SCFAs can also be absorbed in the human SI.^[^ ^33^ ^]^ On the other hand, fermentation in the SI might lead to bloating due to production of gasses in combination with the smaller diameter of the SI compared to the colon.^[^ ^34^ ^]^ Priming and activation of SI bacteria by dietary fibers could potentially result in a better and faster growth when these activated bacteria enter the desired environment in the ascending colon (i.e., lower pH, anaerobic gases). This could lead to more efficient breakdown of fibers in the ascending colon. Our results indicate that GOS and FOS/inulin could have effects in the SI via the residing microbiota, because breakdown started before 5 h of fermentation. Although the time points used in this model cannot be directly translated to the SI transit time, by selection of more extreme, longer incubation times we were able to investigate whether SI microbiota have functionality to degrade dietary fibers. Therefore, to investigate the potential effects of GOS and FOS inside the SI, in vivo studies are needed that capture the short transit time of the SI (median 4.1 h, IQR 3.5–5.9 h)^[^ ^35^ ^]^ and the luminal microbiota of healthy subjects.

Lemon pectin was found to be slowly fermented by SI microbiota of two subjects when compared to the fast GOS and FOS degradation within these subjects, since breakdown started only after 9 h of incubation. An efficient in vitro utilization of pectin by human colonic microbiota was reported before.^[^ ^36^ ^]^ IMMP was found to be slowly fermented by the SI microbiota of subject I5, associated with an increased abundance of *Bacteroides* between 7 and 24 h, whereas the other two subjects did not have the capacity to degrade IMMP. In contrast, all three subjects degraded GOS and FOS. In vitro fermentation with pooled fecal inoculum showed the breakdown of IMMP between 12 and 24 h,^[^ ^10^ ^]^ so also for fecal microbiota IMMP is a slow degradable fiber. Fiber fermentation kinetics varied depending on molecular weight, and sugar and linkage composition, as shown before.^[^ ^36^ ^]^ Different enzymes are required for their degradation. For instance, FOS and inulin can be classified as low‐specificity fibers because many bacteria are able to access and degrade them,^[^ ^37^ ^]^ whereas pectin degradation requires multiple enzymes for degradation. *Bacteroides thetaiotaomicron* is known to use the complex pectic polysaccharides,^[^ ^38^ ^]^ but also for instance *Eubacterium* spp., *Clostridium* spp., and *Bifidobacterium* spp. are pectin‐degraders.^[^ ^39^ ^]^ *Bifidobacterium* and *Clostridium_sensu_stricto_1* were detected in the microbiota of subjects I1 and I2 in this study, and *Bacteroides* spp. were detected previously in the human ileum.^[^ ^40^ ^]^ Breakdown of lemon pectin was related to increased abundance of *Cellulosilyticum* in I2 after 9 h. *Cellulosilyticum* spp. are known to produce pectinases,^[^ ^41^ ^]^ but at the start of fermentation this bacteria was not yet detected. Microbiota from the human ileum mucosa encompasses enzymatic potential for degradation of complex fibers, namely plant cell wall polysaccharides carboxymethylcellulose and xylans, but luminal ileum microbiota was not studied, and this information was based on metagenomics data, which does not capture information about fiber breakdown kinetics.^[^ ^42^ ^]^ The slow fermentation rate of lemon pectin and also IMMP can be explained by the growth of total bacteria, but more likely by growth of specific bacterial groups that produced carbohydrate degrading enzymes required for degradation. Overall, considering the SI transit time in vivo, utilization of lemon pectin and IMMP by the SI microbiota is unlikely. We showed that the metabolism of dietary fibers by SI microbiota is dependent on the molecular structure of the fiber.

The ileostomy effluent samples used in this study contained similar SI bacteria as found before in healthy subjects, for example *Veillonella*, *Streptococcus*, *Lactobacillus, Clostridium cluster_I*, and *Enterococcus*.^[^ ^21^, ^43^, ^44^, ^45^ ^]^ As shown before,^[^ ^43^ ^]^ the SI microbiota profile was highly personal. Fiber fermentation triggered microbiota changes in vitro, which were dependent on the initial microbiota composition of the subject. All subjects were able to degrade GOS and FOS/inulin (DP3‐8) despite the different microbiota profiles, which is not surprising since different bacteria can have similar functions.^[^ ^46^ ^]^ Lactate, known to be an intermediate for the production of acetate, propionate, or butyrate,^[^ ^47^ ^]^ was mainly produced by I4 and I5 upon FOS/inulin and GOS, which could link to the presence of lactate‐producing *Streptococcus* in these subjects.^[^ ^48^ ^]^ In contrast, fiber breakdown was not always associated with specific microbiota changes as observed by a stable Bray‐Curtis dissimilarity over time. For example, I1 degraded lemon pectin after 9 h but no microbiota differences were found compared to the control. The differences in microbiota composition between subjects at baseline can explain why some subjects could degrade a fiber. The personalized microbiota responses to the same fiber substrate were previously confirmed by others for the colonic microbiota in response to food.^[^ ^49^ ^]^ Ultimately, having a personalized nutrition focus with respect to various dietary fibers in future studies may be of interest.

## 4 Concluding Remarks

The applied in vitro batch fermentation enabled us to elucidate SI microbiota functionality with respect to dietary fiber breakdown. Fermentation and degradation kinetics were dependent on the type and size of the fiber. Degradation of prebiotics GOS and FOS by the SI microbiota from all ileostomy subjects was demonstrated. In contrast, the higher molecular weight fibers FOS/inulin DP ≥ 10, lemon pectin, and IMMP showed a slow fermentation rate, exceeding in vivo SI transit time. Acetate was the predominant produced metabolite by the SI microbiota. Microbiota responses in vitro, and consequently metabolite profiles, were dependent on the initial microbiota composition of the individuals, supporting the importance of a personalized nutrition approach that could be especially relevant for dietary fibers.

## 5. Experimental Section

##### Fiber Substrates

The following prebiotic oligo‐ and polysaccharides were used: inulin (DP2‐60) mixed with FOS (DP2‐10) in a 1:1 w/w ratio (Frutafit TEX! and Frutalose OFP; Sensus, Roosendaal, the Netherlands), GOS powder (Vivinal, FrieslandCampina, Wageningen, the Netherlands) composed of approximately 69% GOS, with a DP composition (on weight percentage oligosaccharide) as follows: 31% DP2 (other than lactose), 38% DP3, 18% DP4, 8% DP5, and 5% DP6 or higher, 28% mono‐ and disaccharides and 3% moisture. Furthermore, a lemon pectin with a degree of methyl esterification of 67 (DM67) (CP Kelco, Copenhagen, Denmark), and IMMP with 92% α‐1,6‐linked glycosidic linkages produced from potato starch by the enzyme 4,6‐α‐glucanotransferase, with an average DP of 50^[^ ^50^ ^]^ (AVEBE, Veendam, the Netherlands) were used.

##### Subject Characteristics

Ileostomy effluent was collected from five subjects with an ileostomy bag attached to the distal ileum that were otherwise healthy, and who did not use anti‐, pre‐, or probiotics for at least 3 months before effluent donation. Subjects gave informed consent. The subjects were denoted as subject 1 (I1), subject 2 (I2), etc. Two consecutive days before donation day participants filled in food diaries to determine the total daily fiber intake in the habitual diet, which were analyzed based upon the NEVO table 2016 according to AOAC985.29 (Prosky)^[^ ^51^ ^]^ and AOAC991.43 (Lee)^[^ ^52^ ^]^ methods by dieticians. The ileostomy effluent was collected in the morning after 14 h of fasting, and kept at −20 °C to minimize bacterial activity until use within 9 h after sampling.

##### Small Intestinal Inoculum Preparations

Ileostomy effluent was diluted in standard ileal efflux medium (SIEM) in order to obtain a 1:5 (v/v) sample:SIEM medium ratio. SIEM (modified from^[^ ^53^ ^]^) was obtained from (Tritium Microbiology, Veldhoven, The Netherlands), and adaptations as described elsewhere,^[^ ^54^ ^]^ namely without Tween 80 to avoid interference with apparatus for oligosaccharide measurement, with less carbohydrates (a mixture of pectins, xylan, arabinogalactan, amylopectin and starch, in total 0.24 g L^−1^), and with MgSO_4_ (0.8 g L^−1^). The pH of the medium was set at seven using a 1 m 2‐(n‐morpholino)ethanesulfonic acid buffer, based on pH measurements in the ileum of ileostomates and healthy adults.^[^ ^21^, ^35^ ^]^ The diluted samples were filtered using a sieve with 1.6 mm holes to remove large food particles, and afterwards used for preselection of the ileostomy effluent in SIEM for 15 h under anoxic conditions (37 °C, shaking at 100 rpm)^[^ ^28^, ^55^ ^]^ for removal of left over carbohydrates in the inoculum.

##### In Vitro Batch Fermentation

The fermentation bottles were filled under anoxic conditions (81% N_2_, 15% CO_2_, and 4% H_2_) with preselected SI inoculum, and SIEM containing the fibers of interest, in volume ratio 1:2. At the start of fermentation, the mixture consisted of 10% of original SI sample in SIEM and 10 g L^−1^ added dietary fibers. The SI inoculum without added fiber was included as control to monitor background fermentation. Fibers without SI inoculum were included to check for contamination. FOS/inulin, and GOS were tested with five subjects I1‐I5. Additionally, in an explorative way two high molecular weight fibers were included; lemon pectin was tested with two subjects (I1‐I2), and in subsequent fermentation experiments (based on the date of ileostomy effluent donation, and consequently the date of the experiment) IMMP, was tested with three other subjects (I3‐I5). Fermentations of different fibers using fresh effluent from one subject were always performed on the same date. Duplicated fermentation bottles were closed by a rubber cap and metal ring in an anaerobic chamber. Incubation took place in duplicate at 37 °C with continuously shaking at 100 rpm. Since slow or no fiber fermentation by SI microbiota was expected, samples were taken at 0 h, and after 5, 7, 9, and 24 h of incubation, using a 2.5 mL syringe with a 0.8 mm × 50 mm needle. Aliquots were directly frozen in liquid nitrogen and stored at −80 °C until analysis.

##### Molecular Weight Distribution of Polysaccharides and Oligosaccharide Profiling

Molecular weight distribution of lemon pectin was analyzed by high performance size exclusion chromatography (HPSEC, Ultimate 3000 HPLC, Dionex, Sunnyvale, CA, USA) with refractive index (RI, Showa Denko, Tokyo, Japan) detection. Samples were centrifuged (10 min, RT, 15 000 × *g*), and supernatant was diluted with demineralized water to a maximum concentration of 5 mg mL^−1^ fiber before analysis. The analysis was performed as described elsewhere.^[^ ^56^ ^]^ Samples (10 µL) were eluted with NaNO_3_ (0.2 m, flow rate 0.6 mL min^−1^, at 55 °C). Mono‐ di‐ and oligosaccharides profiles of GOS, FOS/inulin, and IMMP were analyzed by high performance anion exchange chromatography (HPAEC, Dionex) with pulsed amperometric detection (PAD, ICS5000 ED, Dionex). Samples were centrifuged (10 min, RT, 15 000 × *g*), and supernatant was diluted with demineralized water to a fiber concentration of 0.5 mg mL^−1^ before analysis. The analysis was performed as described elsewhere.^[^ ^57^ ^]^ Peak areas between different fermentation time points were calculated and expressed as percentage present of the initial fiber.

##### Production of Microbial Fermentation Products

Acetate, butyrate, propionate were analyzed by gas chromatography (GC TRACE 1300, Thermo Scientific) with a flame ionisation detector (Interscience, Breda, the Netherlands), equipped with a GC capillary column (25 m × 0.53 mm × 1.00 µm, Agilent CP‐FFAPCB for free fatty acids, Varian‐Chrompack), details are provided in the Supporting Information. Formate, lactate, and succinate were analyzed using HPLC UltiMate 3000 system with a Shodex RI‐101 detector (Dionex, Sunnyvale, CA, USA). The fermentation samples were centrifuged before use (10 min, RT, 15 000 × g). The supernatant was diluted four times with demineralized water. The methods, column, guard column, and software were used as described elsewhere.^[^ ^58^ ^]^

##### Microbiota Profiling

Bacterial DNA was extracted from 300 µL fermentation samples, or from 0.25 g ileostomy effluent. Bacterial cell lysis was achieved by a repeated bead beating method, in combination with ASL Stool lysis buffer (Qiagen, Hilden, Germany) as described previously.^[^ ^59^ ^]^ The obtained lysate was used for DNA extraction and purification using the QIAamp DNA Mini Kit (Qiagen). Triplicate PCR reactions were performed using barcoded primers F784‐R1064.^[^ ^60^, ^61^ ^]^ Microbiota composition was determined via sequencing of the 16S rRNA gene using the variable region V5–V6 on the Illumina HiSeq2500 platform (Eurofins GATC Biotech, Konstanz, Germany). Raw sequencing data was processed and amplicon sequence variants (ASV) were picked with NG‐Tax using default settings.^[^ ^61^, ^62^ ^]^ Taxonomy was assigned with the SILVA database (version 128). Further details of this procedure are provided in Supporting Information. R version 3.5.1. was used for all analyses.^[^ ^63^ ^]^ Before analysis, contaminants were removed and quality checks were carried out (Supporting Information). Alpha‐diversity was calculated using the inverse Simpson diversity index. Beta‐diversity was calculated by Bray–Curtis dissimilarity on relative bacterial abundances to determine overall microbiota differences between groups and visualized using Principal Coordinate Analysis (PCoA). Permutational multivariate analysis of variance (PERMANOVA) with post‐hoc testing was used to determine differences in overall community composition between subject, fibers, and time. In the heatmap samples were hierarchically clustered using Bray‐Curtis dissimilarity and the Ward.D2 agglomeration algorithm. Details of used packages, and settings of the analyses are described in Supporting Information.

##### Bacterial 16S rRNA Gene Copy Number Quantification

The total bacterial abundance was determined by amplifying a conserved region of the 16S rRNA gene (in between the V8 and V9 region) with q‐PCR. The PCR reaction mixture contained SensiMix (Bioline, GC biotech, Alphen aan den Rijn, Netherlands), the primers 1369F and 1492R (100 µm), and 2 µL of diluted genomic DNA (5 ng µL^−1^). Apparatus, primers, and PCR cycling conditions were used as described elsewhere.^[^ ^64^ ^]^

##### Statistical Analysis

Statistical analysis of SCFAs was performed on probabilistic quotient normalization transformed data.^[^ ^65^ ^]^ Linear mixed models in R were used for assessment of fiber and time effects on the SCFA concentrations, using the lme4 package.^[^ ^66^ ^]^ Fiber type, time, and their interactions were included as fixed effects, subjects as a random effect with time added as a random slope. A *p*‐value < 0.05 was considered significant.

## Conflict of Interest

The authors declare no conflict of interest.

## Author Contributions

The author's responsibilities were as follows – G.J.E.J.H. and H.S. conceived the study; M.v.T., C.R., H.S., G.J.E.J.H. designed the study; M.v.T., M.L., C.R., S.K., R.A. conducted/assisted with the experimental work; R.A., G.H., and E.Z. assisted microbiological analyses; M.v.T. prepared the first manuscript draft, which was revised by all co‐authors. All authors read and approved the final manuscript.

## Supporting information



Supporting information

## Data Availability

The data that supports the findings of this study are available in the supplementary material of this article.
