# Supplementary material for: Fermentation Kinetics of Selected Dietary Fibers by Human Small Intestinal Microbiota Depend on the Type of Fiber and Subject
Source: Mol Nutr Food Res. 2020 Sep 29;64(20):2000455. doi: 10.1002/mnfr.202000455 (PMC7685165; doi:10.1002/mnfr.202000455)
Supplement: Supplementary file 1 — Supporting information [file MNFR-64-2000455-s001.docx]

**Fermentation kinetics of selected dietary fibers by human small intestinal microbiota depend on the type of fiber and subject**
Mara P.H. van Trijp, Christiane Rösch, Ran An, Shohreh Keshtkar, Madelon J. Logtenberg, Gerben D.A. Hermes, Erwin G. Zoetendal, Henk A. Schols, Guido J.E.J. Hooiveld

**Supporting Methods**

**Production of microbial fermentation products**

Before analysis of SCFAs, the fermentation samples were centrifuged (10 min, RT, 15000 x g). 100 µL supernatant was mixed with 50 µL solution containing HCl (0.3 M), oxalic acid (0.09 M), and internal standard 2-ethyl butyric acid (0.45 mg/mL). Samples were vortexed, incubated 30 min at room temperature, and centrifuged (5 min, RT, 15000 x g). The GC oven program was as follows: 100°C for 0.5 min, raised to 180°C (8°C/min) hold for 1 minute, raised to 200°C (20°C/min) and hold for 5 min. The split flow was 40 mL/min. Helium was used as carrier gas. Data was analyzed with Thermo Xcalibur software version 2.2.

**Library preparations and microbiota sequencing**

First, the extracted DNA was diluted to 20 ng/µL. PCR reactions were performed in 35 µL triplicate reactions, containing 7 µL 5x Phusion Green HF buffer, 0.7 µL 10 mM dNTPs (Promega), 0.4 µL Phusion hot start II DNA polymerase (2U/µL), 25.5 µl nuclease free water, 0.7 µL of template DNA (20 ng/µL) and 0.7 µL of each of the barcoded primers F784-R1064 (10 µM). Cycling conditions were as follows: 98°C 30 sec, 25 cycles of 98°C 10 sec, 42°C 10 sec, 72°C 10 sec, and 72°C for 7 minutes. Pooled PCR products were checked for correct size on a 1.3% agarose gel, and subsequently purified using magnetic beads (MagBio Genomics Inc., Gaithersburg, USA). PCR products were quantified using Qubit dsDNA BR buffer and dye (Invitrogen, California, USA), on a Qubit 2.0 Fluorometer. Afterwards, a library containing an equimolar mix (200 ng each) of purified PCR products was prepared. In total, 136 fermentation samples, 2 theoretical mock controls, and 2 negative controls (DNA isolated from nuclease free water, and a negative control from the fermentation experiment) were prepared. The resultant libraries were concentrated by using magnetic beads, and sequenced on the Illumina HiSeq2500 platform (Eurofins GATC Biotech, Konstanz, Germany).

**Sequencing data processing**

Raw sequencing data was processed using NG-Tax version 1.0 with default settings.^[1-2]^ In short, libraries were quality checked by selecting reads with perfect matching primer sequences and de-multiplexed by selecting read pairs with perfectly matching valid barcodes. Amplicon sequence variants (ASV), which is an individual sequence variant, were picked as follows: sequences were ordered by abundance per sample and reads were considered valid when their cumulative abundance was ≥0.1%. The SILVA reference database version 128 was used to assign taxonomy, with a confidence of >80% for genus level classification. Contaminants were removed based on their abundance in the negative extraction control, and being flagged previously in literature as laboratory reagent contaminants.^[3]^ The following genera were removed: *Nesterenkonia, Ralstonia, Epulopiscium, Trichococcus*, and *Caldalkalibacillus*. These bacteria were present in low abundance only in biological samples with a low input DNA concentration. Quality control was performed by calculating pairwise Pearson correlations, between the known composition of the control and sequenced positive controls (mock), and within technical and biological replicates using genus level relative abundance. Pearson correlations were also calculated for pairwise combinations of microbiota composition using genus level relative abundance in ileostomy effluents from different subjects.

**Microbiota profiling**

Raw counts were transformed to relative abundance. The alpha-diversity (within sample diversity) was calculated using inverse Simpson diversity index using the microbiome package.^[4]^ Beta-diversity (between sample diversity) was used to determine overall microbiota differences between groups. To visualize the microbiota variation, a principal coordinate analysis (PCoA) was performed using Bray-Curtis as implemented in the phyloseq package.^[5]^ Permutational multivariate analysis of variance (PERMANOVA) as implemented in the vegan package ^[6]^ was used to determine whether there were differences using Bray-Curtis in overall community composition between subjects, fibers, and time. Sample-wise distances were calculated within individual for fiber samples versus control samples at each time point using Bray-Curtis. Heatmaps were generated using pheatmap ^[7]^, scaled per row and hierarchically clustered using the Ward.D2 algorithm. Fermentation samples were clustered using Bray-Curtis dissimilarity.

**Supporting Results**

**Amplicon sequencing quality control**

The average number of reads per sample was 182736±78324. In total 884 unique ASV were identified in the microbiota dataset, within 89 unique genera. One taxon was removed after mitochondrial filtering. To check for the effect of sequencing depth on diversity we correlated an alpha diversity metric with the number of reads and found no correlation (**Figure S1 A**). To test for the reproducibility of the sequencing two synthetic samples of known composition were sequenced (mock communities).^[2]^ Pearson correlations of the theoretical composition with the sequenced results were 0.81 for Mock 3 in both sequencing libraries, and 0.83 (library 1) and 0.87 (library 2) for Mock 4. Pearson correlations between 24 biological duplicates were 0.972±0.04 (Figure S1 B). Pearson correlations of five technical duplicates (same DNA sample, but different PCR barcode) were between library 0.994±0.001, and within library 0.985±0.018 (Figure S1 C).

**Microbiota in the ileostomy effluent samples**

All ileostomy effluents contained members of genera *Streptococcus* and *Clostridium cluster I*, and the family Peptostreptococcaceae and Enterobacteriaceae. In addition, also genera such as *E.eligens*, *Veillonella*, *Lactobacillus*, *Enterococcus*, *Haemophilus*, *Bifidobacterium*, *Terrisporobacter*, *Klebsiella*, *Turicibacter*, and *Escherichia-Shigella*, were detected (**Figure S2**).

**Table S1.** Characteristics of the ileostomy subjects.

| Participant | Gender | Age (years) | BMI (kg/m^2^) | Ileostomy wearing time (years) | Reason for ileostomy | Medication^a)^ |
| --- | --- | --- | --- | --- | --- | --- |
| 1 | Female | 71 | 14.9 | 14 | Damaged colonic epithelial layer | Domperidon, magnesium hydroxide, electrolytes |
| 2 | Female | 75 | 29.7 | 3 | Colon cancer | Valsartan |
| 3 | Female | 30 | 20.1 | 1 | Ulcerative colitis | None |
| 4 | Male | 32 | 19.6 | 7 | Ulcerative colitis | Desloratadine |
| 5 | Male | 58 | 21.2 | 7 | Ulcerative colitis | None |

^a)^ Domperidon, dopamine antagonist used to stimulate peristaltic movements; magnesium hydroxide, laxative; electrolytes, used against constipation; valsartan, angiotensin II receptor antagonist used to lower blood pressure; desloratadine, anti-histamine allergy drug.

| Subject | Day | Energy (kcal) | Total protein (energy%) | Total fat (energy%) | Total carbohydrates  (energy%) | Total dietary fibers  (energy%) | Total carbohydrates^a)^ (gram) | Total dietary fibers^b)^ (gram) | Total alcohol (gram) |
| --- | --- | --- | --- | --- | --- | --- | --- | --- | --- |
| 1 | 1 | 1611 | 22.9 | 32.0 | 41.9 | 3.3 | 166.5 | 27.9 | 0.0 |
|  | 2 | 1502 | 18.4 | 20.3 | 51.0 | 3.7 | 189.3 | 29.0 | 14.8 |
| 2 | 1 | 2062 | 14.8 | 34.1 | 48.2 | 1.5 | 245.6 | 16.2 | 0.0 |
|  | 2 | 2199 | 18.2 | 26.7 | 51.7 | 2.0 | 281.4 | 22.9 | 0.0 |
| 3 | 1 | 1468 | 15.7 | 28.5 | 53.0 | 2.3 | 192.0 | 17.5 | 0.0 |
|  | 2 | 898 | 13.3 | 28.2 | 55.1 | 3.0 | 122.6 | 14.4 | 0.0 |
| 4 | 1 | 3275 | 14.3 | 37.4 | 44.9 | 0.8 | 363.0 | 14.6 | 12.5 |
|  | 2 | 2057 | 9.9 | 34.3 | 54.9 | 1.0 | 279.9 | 11.1 | 0.0 |
| 5 | 1 | 2619 | 20.0 | 42.9 | 32.7 | 1.3 | 210.9 | 18.1 | 10.0 |
|  | 2 | 2292 | 16.8 | 39.8 | 41.0 | 2.0 | 231.4 | 23.5 | 0.0 |

**Table S2.** Information about the dietary intake (energy, fat, carbohydrates, fibers, alcohol) of the five subjects on the two days consecutive to ileostomy effluent donation.

The five ileostomy subjects are numbered from 1 to 5. 24-hour food diaries kept the two days consecutive to ileostomy sample donation.
^a)^Total carbohydrates without dietary fibers.
**^b)^**Included in dietary fibers are high molecular weight dietary fiber (e.g. cellulose, resistant starch, cereal β-glucan, guar gum and certain xylans), insoluble dietary fiber in water (e.g. cellulose, resistant starch and certain xylans), dietary fiber soluble in water and precipitated by 78% ethanol (e.g. cereal β-glucan, guar gum and certain xylans). Excluded are low molecular weight dietary fiber (e.g. FOS, GOS, a portion of Polydextrose®, inulin and resistant maltodextrins) and non-resistant starch.

**Figure S1.**

*
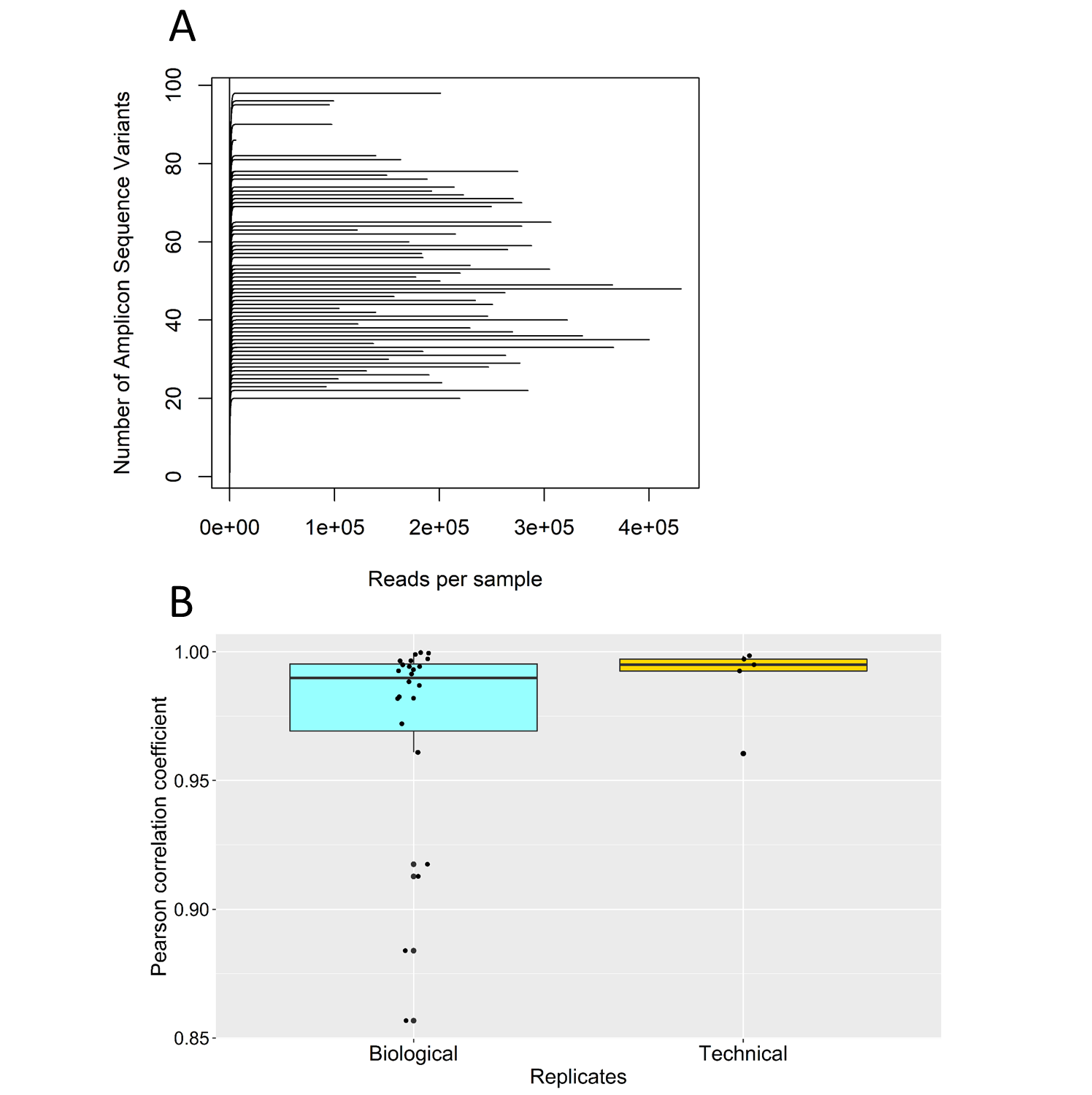
*

Quality characteristics of the amplicon sequencing data. The rarefaction curve of all samples in the *in vitro* fermentation experiment (A), Pearson correlation coefficients of biological replicates pairs and technical replicate pairs including two mock positive controls (B). Boxplots show the distribution of the data via quartiles.

**Figure S2.**


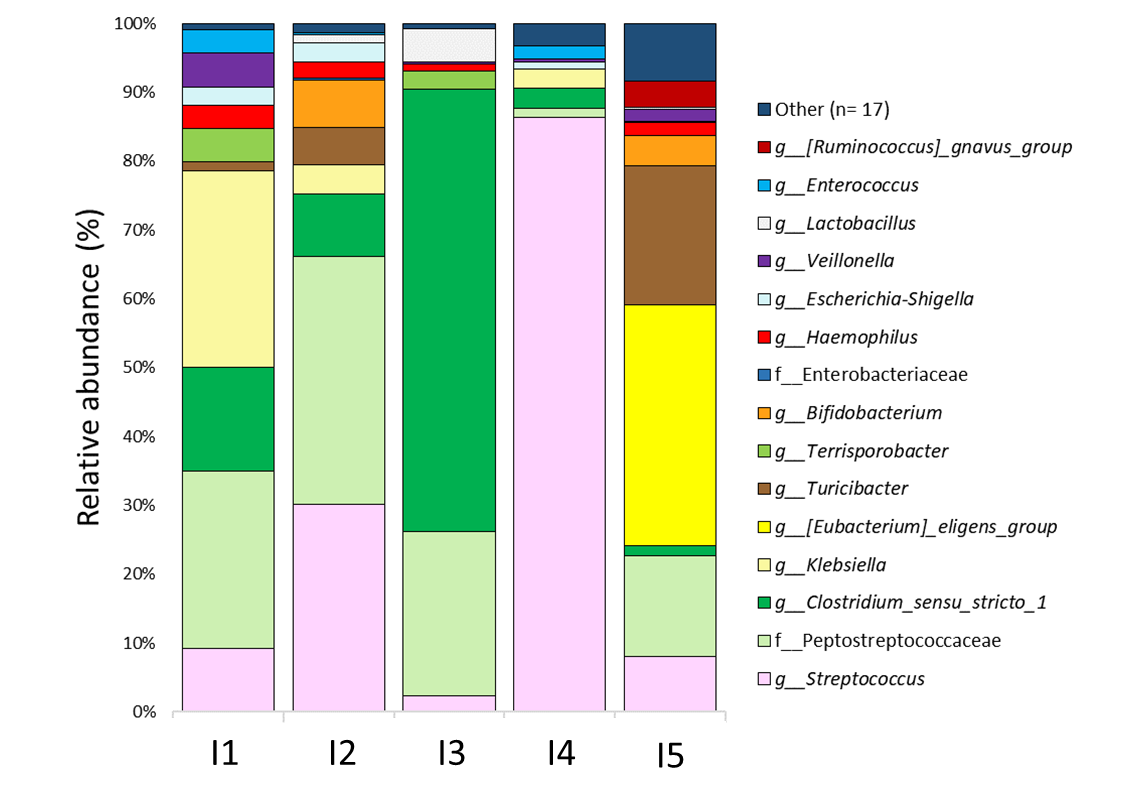


The ileostomy effluent samples obtained from five subjects (I1-I5). The relative abundances of the 15 most abundant genera are shown. If genera was not classified or the genus was an uncultured bacteria, only the family level is depicted in the graph.

**Figure S3.**

Changes in pH values in the in vitro fermentation samples of the five subjects: I1 (A), I2 (B), I3 (C), I4 (D), and I5 (E) for the different dietary fibers at 0, 5, 7, 9, and 24 h and for the control without added fiber. Values are means of replicates ± SD. FOS, fructo-oligosaccharides; GOS, galacto-oligosaccharides; IMMP, isomalto-maltopolysaccharides.

**Figure S4.**

**
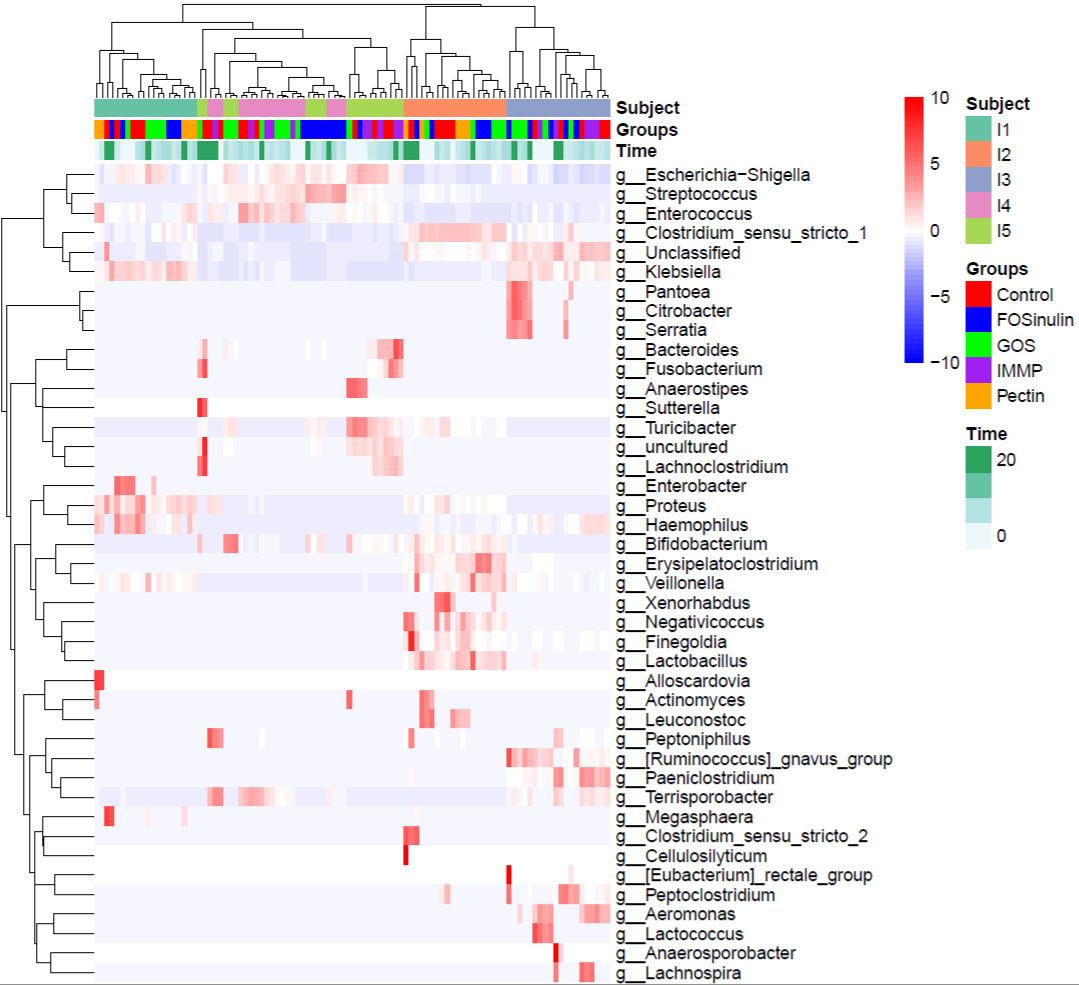
**

A heatmap of all *in vitro* fermentation samples, to visualize taxa causing differences between subjects and fibers. Each column represents a sample, each row a unique genera. On top of the heatmap diverse colors depict the five subjects, fibers, and time points. Fermentation samples were clustered on Bray-Curtis microbial dissimilarity, and genera were hierarchically clustered using the Ward.D2 algorithm. Colors in the heatmap were given by taxa scaling, with the mean relative abundance set at 0, and taxa relative abundances above the mean in red, taxa relative abundances below the mean in blue. White means the taxa was not detected in the sample. FOS, fructo-oligosaccharides; GOS, galacto-oligosaccharides; IMMP, isomalto/malto-polysaccharide; I, ileostomy inoculum.

**Figure S5.**


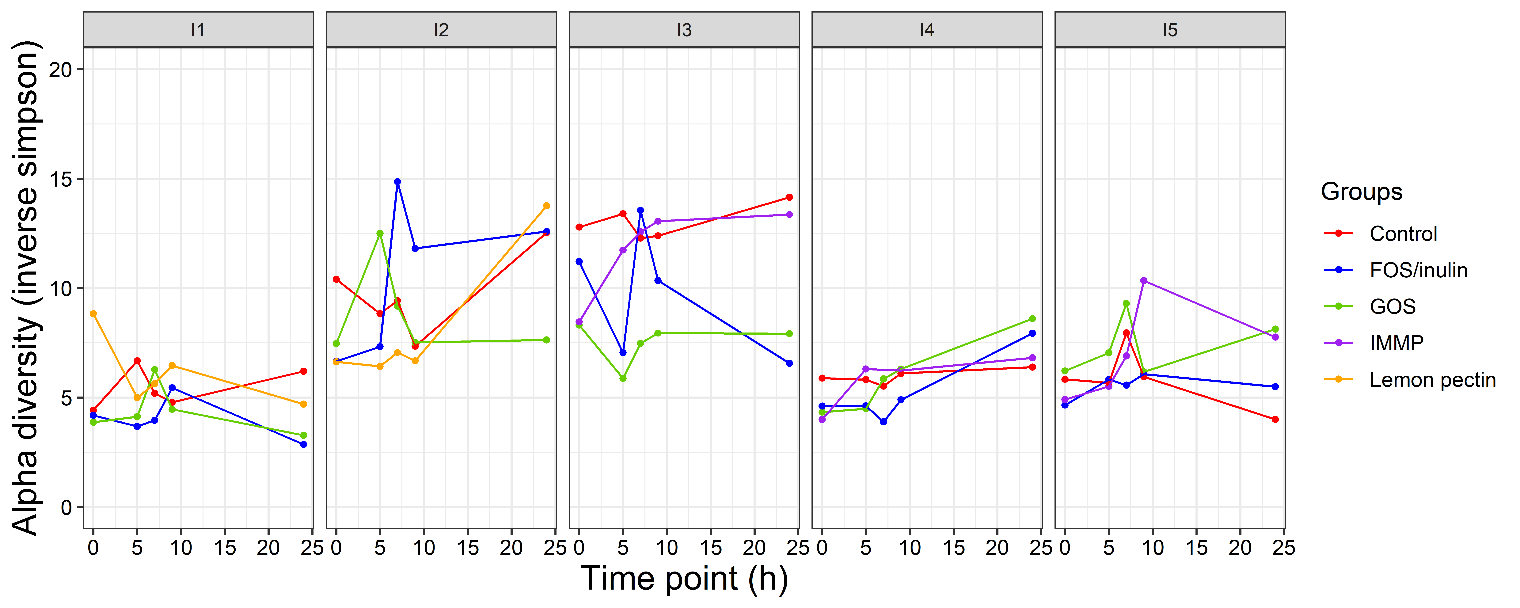
Alpha-diversity over time in the *in vitro* fermentation samples of the five subjects (I1-I5). The lines depict the alpha-diversity during fermentation with different fibers, or control without added fiber. FOS, fructo-oligosaccharides; GOS, galacto-oligosaccharides; I, ileostomy inoculum; IMMP, isomalto/malto-polysaccharides.

**Figure S6.**

**
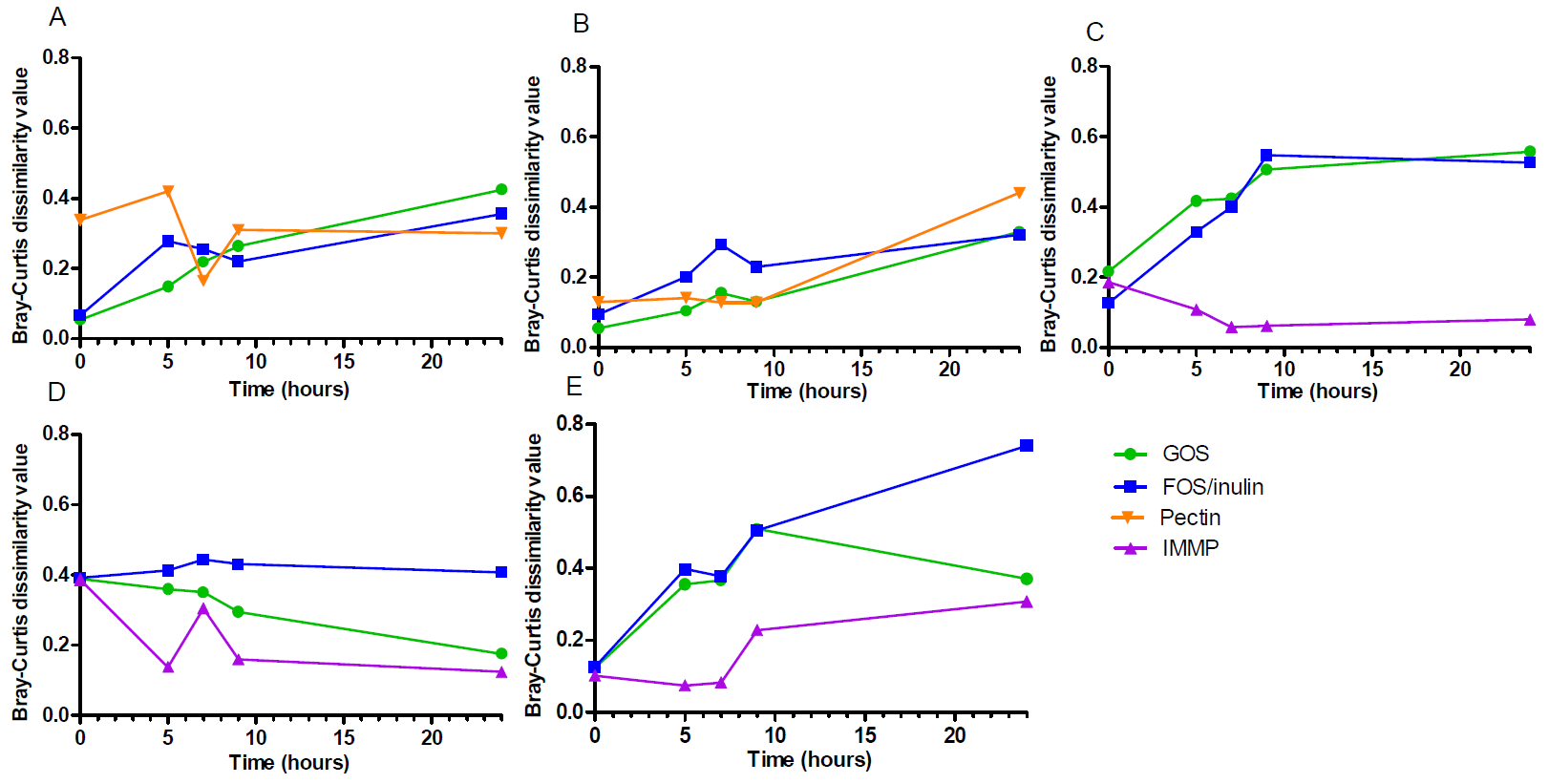
**

Bray-Curtis dissimilarity values were calculated for the fibers compared to control without added fiber at 0, 5, 7, 9, and 24 h during *in vitro* fermentation, within each of the five subjects: I1 (A), I2 (B), I3 (C), I4 (D), and I5 (E). FOS, fructo-oligosaccharides; GOS, galacto-oligosaccharides; IMMP, isomalto/malto-polysaccharide.

**Figure S7.**


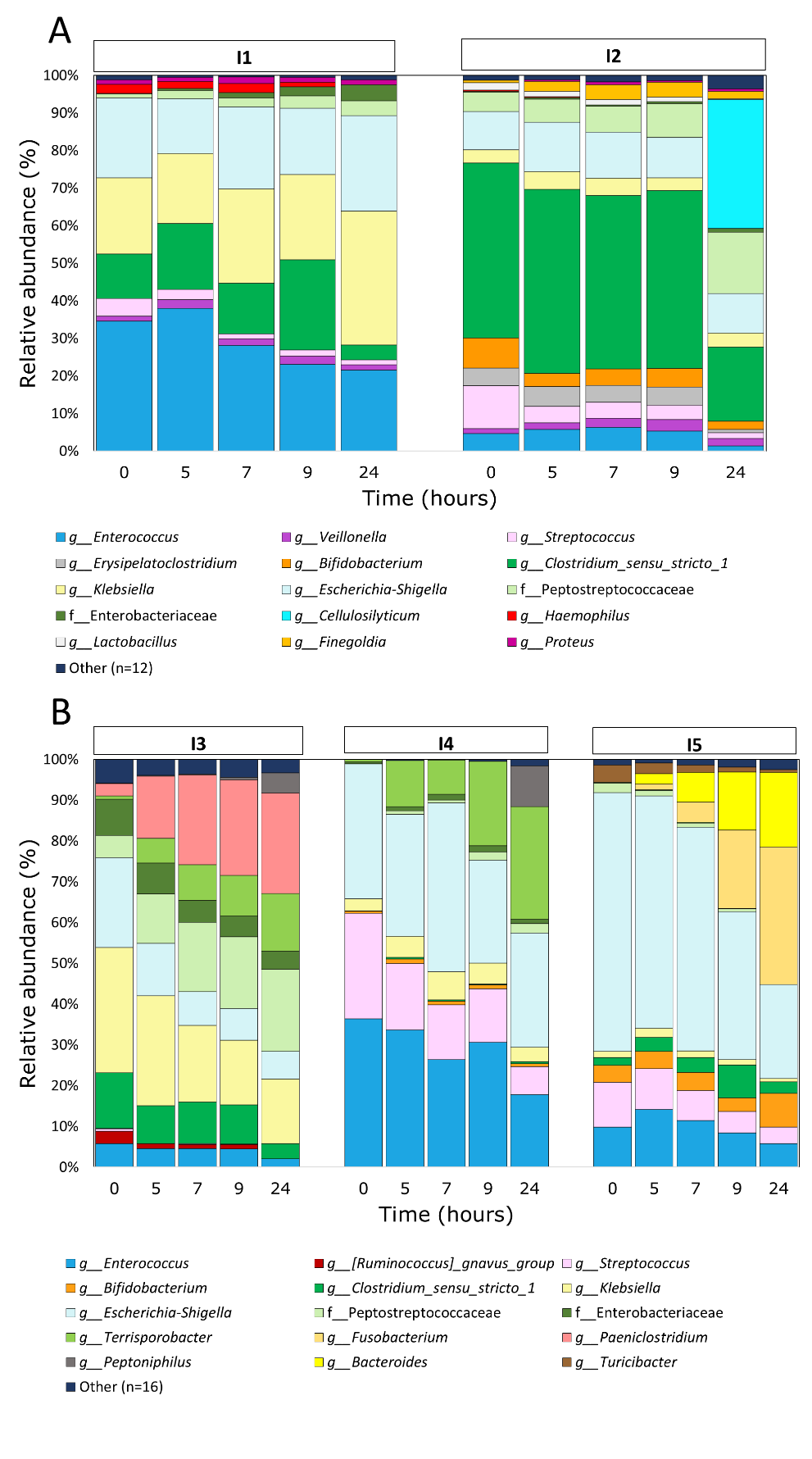


Relative abundance differences of the top 15 genera (or highest known taxonomy) at 0, 5, 7, 9, and 24 h during *in vitro* fermentation of lemon pectin (A) by two subjects I1, I2, and during IMMP (B) fermentation by three subjects I3, I4, and I5. I, ileostomy inoculum.

**Figure S8.**

**
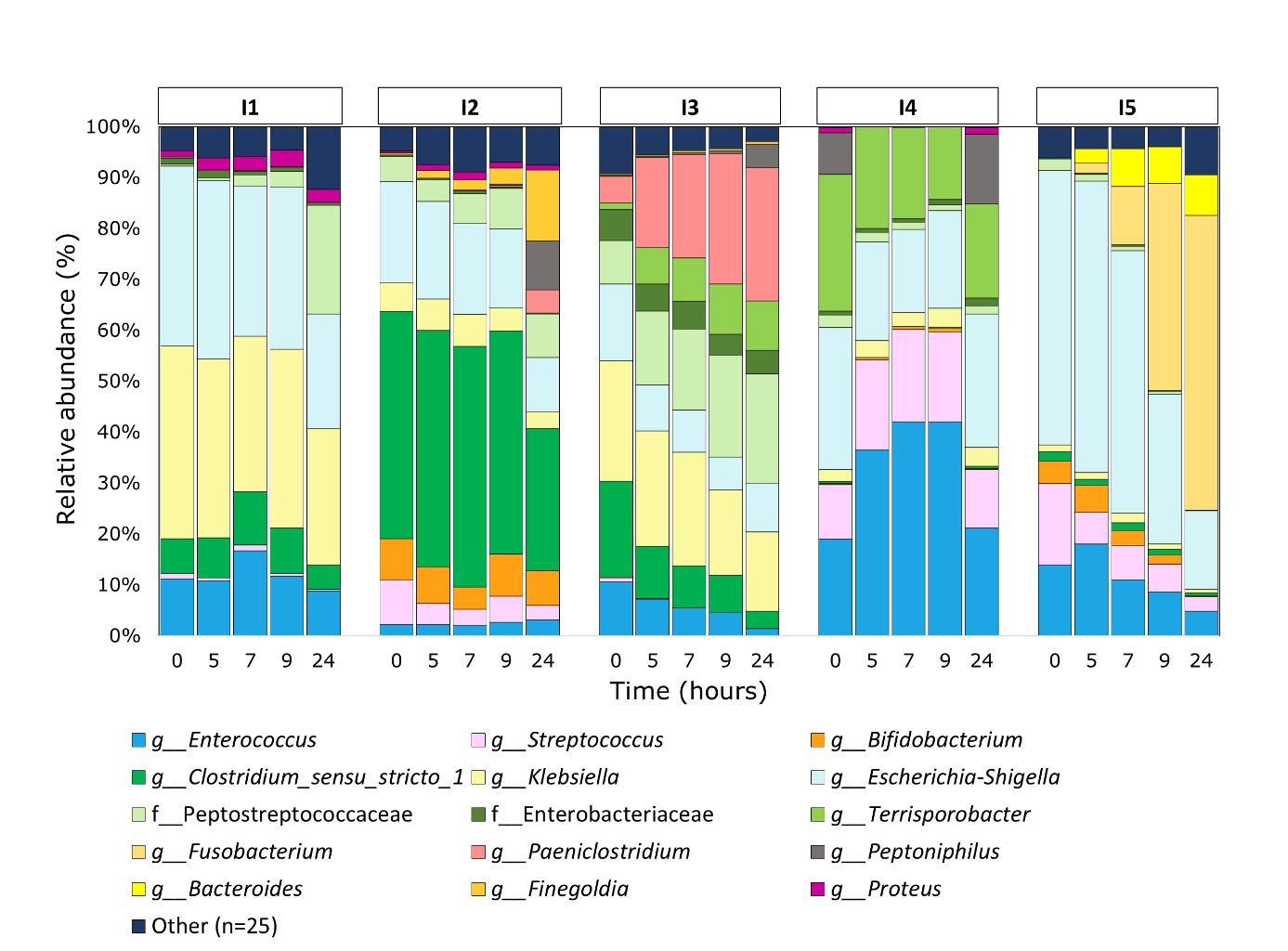
**

Relative abundance differences of the top 15 genera (or highest known taxonomy) at 0, 5, 7, 9, and 24 h during *in vitro* fermentation with control without fiber by five subjects I1-I5 (A). I, ileostomy inoculum.

**Figure S9.**

Changes of total 16S rRNA copy numbers during in vitro fermentation of different fibers or for the control without added fiber at 0, 5, 7, 9, and 24 h for the five subjects I1 (A), I2 (B), I3 (C), I4 (D), and I5 (E), as indications of changes in total bacteria presence. Values are means ± SDs. FOS, fructo-oligosaccharides; GOS, galacto-oligosaccharides; IMMP, isomalto-maltopolysaccharides; rRNA, ribosomal RNA; RS, resistant starch.

**References**

[1] W. Poncheewin, G.D.A. Hermes, J.C.J. van Dam, J.J. Koehorst, H. Smidt, P.J. Schaap, *Front Genet* **2019**, *10*, 1366.

[2] J. Ramiro-Garcia, G. Hermes, C. Giatsis, D. Sipkema, E. Zoetendal, P. Schaap, H. Smidt, *F1000Research* **2016**, *5*,

[3] S.J. Salter, M.J. Cox, E.M. Turek, S.T. Calus, W.O. Cookson, M.F. Moffatt, P. Turner, J. Parkhill, N.J. Loman, A.W. Walker, *BMC Biology* **2014**, *12*, 87.

[4] S.S.e.a. Leo Lahti, Tools for microbiome analysis in R. Microbiome package version 1.5.25. , Bioconductor **2017**.

[5] P.J. McMurdie, S. Holmes, *PLOS ONE* **2013**, *8*, e61217.

[6] F.G.B. Jari Oksanen, Michael Friendly, Roeland Kindt, Pierre Legendre, Dan McGlinn, Peter R. Minchin, R. B. O'Hara, Gavin L. Simpson, Peter Solymos, M. Henry H. Stevens, Eduard Szoecs and Helenev Wagner, vegan: Community Ecology Package. R package version 2.5-3., **2018**.

[7] R. Kolde, pheatmap: Pretty Heatmaps. R package version 1.0.10., **2018**.
